# P-1067. *In Vitro* Potency of Ceftibuten-Avibactam (CTB-AVI) and Comparators against Extended-Spectrum Beta-Lactamase (ESBL)-like Enterobacterales from Outpatient Centers and Nursing Homes in the United States

**DOI:** 10.1093/ofid/ofae631.1255

**Published:** 2025-01-29

**Authors:** Aliaa Fouad, April M Bobenchik, Andrew E Clark, Lars Westblade, Holly K Huse, Isabella W Martin, A Brian Mochon, Erik Munson, Maroun M Sfeir, Monica Srodon, Yungchou Wang, David P Nicolau, Tomefa E Asempa

**Affiliations:** Hartford Hospital, Farmington, Connecticut; Penn State Milton S. Hershey Medical Center, Hershey, Pennsylvania; University of Texas Southwestern Medical Center, Dallas, Texas; Weill Cornell Medicine, New York, NY; Harbor-UCLA Medical Center, Torrance, California; Dartmouth-Hitchcock Medical Center, Lebanon, NH; University of Arizona College of Medicine, Phoenix, Arizona; Marquette University, Milwaukee, Wisconsin; University of Connecticut Health Center, Farmington, Connecticut; Eastern Connecticut Health Network, Manchester, Connecticut; Cape Regional Health System, Cape May Court House, New Jersey; Hartford Hospital, Farmington, Connecticut; Hartford Hospital, Farmington, Connecticut

## Abstract

**Background:**

CTB is a currently available oral third-generation cephalosporin that is being developed in combination with the oral prodrug of AVI. In this study, we evaluated the *in vitro* activity of CTB-AVI against clinical ESBL-like Enterobacterales that were collected from outpatient settings.

**Methods:**

156 ESBL-like Enterobacterales isolates were received from 10 US medical centers that processed cultures from affiliated outpatient centers in 2022. Susceptibility tests for CTB-AVI and standard-of-care antibiotics were performed by CLSI broth microdilution method. Isolates were designated ESBL-like based on cefpodoxime MIC ≥2 mg/L for *Proteus mirabilis* and ceftriaxone MIC ≥2 mg/L for all other Enterobacterales. Fosfomycin disk diffusion was performed on *E. coli* isolates only and interpreted according to CLSI M100Ed32 guidance. CTB-susceptible breakpoints currently published by CLSI (≤8 mg/L) and EUCAST (≤1 mg/L) were applied to CTB-AVI for comparison.

**Results:**

Isolates were primarily *E. coli (*60%) and *Klebsiella pneumoniae (*16%) and were recovered from patients seen in nursing home/long-term care (38%), urology (21%), and ambulatory care (19%) clinics. Based on CLSI and EUCAST breakpoints, CTB had susceptibility rates of 66% and 32%, respectively. The addition of AVI (fixed concentration of 4 mg/L) to CTB resulted in *in vitro* potentiation, resulting in 99% (CLSI) and 97% (EUCAST) susceptibility. Susceptibility rates of all isolates against comparator antimicrobials were as follows: cefpodoxime (7%), ceftriaxone (2%), ceftazidime/avibactam (CAZ-AVI) (99.4%), tebipenem (86% at preliminary susceptible breakpoint ≤0.125 mg/L), ertapenem (92.3%), levofloxacin (29%), sulfamethoxazole/trimethoprim (SMX-TMP) (47.4%), and fosfomycin (96%). CTB-AVI had 4-fold lower MIC_50/90_ values relative to CAZ-AVI (MIC_50/90_; 0.031/0.25 versus 0.25/1 mg/L). Overall, 100% of levofloxacin and SMX-TMP co-resistant isolates (n=43) were inhibited at CTB-AVI MIC breakpoint concentrations of 8 and 1 mg/L.

**Conclusion:**

These data demonstrate potent *in vitro* activity of CTB-AVI against ESBL-like Enterobacterales isolated from patients in US nursing homes and outpatient clinics warranting further studies this oral treatment option.

**Disclosures:**

**April M. Bobenchik, PhD**, Becton Dickinson: Grant/Research Support|BioMerieux: Advisor/Consultant|BioMerieux: Grant/Research Support|BioMerieux: Honoraria|Q-Linea: Grant/Research Support|QuidelOrtho: Grant/Research Support|TechLab: Grant/Research Support **Lars Westblade, PhD**, Accelerate Diagnostics, Inc: Grant/Research Support|bioMerieux, Inc: Grant/Research Support|Element Materials Technology: Grant/Research Support|Hardy Diagnostics: Grant/Research Support|Roche Molecular Systems, Inc.: Advisor/Consultant|Roche Molecular Systems, Inc.: Grant/Research Support|Selux Diagnostics, Inc.: Grant/Research Support|Shionogi, Inc: Advisor/Consultant|Talis Biomedical: Advisor/Consultant **Erik Munson, PhD**, Hologic, Incorporated: Advisor/Consultant|Hologic, Incorporated: Honoraria **David P. Nicolau, PharmD**, CARB-X: Grant/Research Support|Innoviva: Grant/Research Support|Innoviva: Honoraria|Merck: Advisor/Consultant|Merck: Grant/Research Support|Merck: Honoraria|Pfizer: Advisor/Consultant|Pfizer: Grant/Research Support|Pfizer: Honoraria|Shionogi: Advisor/Consultant|Shionogi: Grant/Research Support|Shionogi: Honoraria|Venatorx: Grant/Research Support **Tomefa E. Asempa, PharmD**, FDA/CDER: Grant/Research Support|Paratek: Grant/Research Support|Shionogi: Grant/Research Support|Spero: Grant/Research Support|VenatoRx: Grant/Research Support

